# Case Report: Intestinal necrosis secondary to internal abdominal hernia in a young female

**DOI:** 10.3389/fsurg.2026.1876453

**Published:** 2026-07-21

**Authors:** Rui Zhang, Yuhang Yin, Guangrong Gao, Di Xin, Cheng Zhang, Xingshun Qi

**Affiliations:** 1 Department of Gastroenterology, General Hospital of Northern Theater Command, Shenyang, China; 2 Postgraduate College, China Medical University, Shenyang, China; 3 Department of General Surgery, General Hospital of Northern Theater Command, Shenyang, China; 4 Department of Diagnostic Radiology, General Hospital of Northern Theater Command, Shenyang, China

**Keywords:** internal abdominal hernia, intestinal obstruction, multidisciplinary therapy, surgery, timing

## Abstract

Intestinal obstruction and necrosis are emergent and potentially lethal. They are rarely secondary to internal abdominal hernia, which refers to the protrusion of a visceral organ or tissue from its normal anatomical location into another compartment within the abdominal cavity through a congenital or acquired anatomical defect, such as a weakness, opening, or foramen. Its contents are predominantly composed of the stomach and intestinal loops. Internal abdominal hernia occurs suddenly, rapidly progresses, and frequently leads to secondary intestinal ischemia, as well as serious complications, such as intestinal strangulation and necrosis, with a high mortality. Therefore, it highlights the significance of early recognition and timely intervention for improving patient outcomes. In this paper, we reported a case of intestinal obstruction and necrosis secondary to internal abdominal hernia, which was successfully treated by surgery, in spite of a delayed diagnosis.

## 1 Case presentation

On August 6, 2024, a 20-year-old female was transferred to our hospital due to persistent abdominal pain for 26 days and hematochezia for 8 days. On July 11, 2024, she developed acute cramping pain in the upper abdomen, accompanied by abdominal distension at local hospital. Unenhanced abdominal CT scans indicated intestinal obstruction. Diagnostic paracentesis revealed hemorrhagic ascites, indicating a diagnosis of extensive bowel necrosis and peritonitis. It was recommended to perform emergency exploratory laparotomy, but the patient's family strongly declined surgical intervention due to their concerns about surgical risks. One hour later, the patient developed septic shock. After anti-shock treatment, the patient's symptoms improved. On July 29, 2024, the patient developed hematochezia, which was persistent despite symptomatic treatment. Notably, at that time, physical examination showed abdominal distension without any obvious signs of peritoneal irritation.

**Figure 1:**
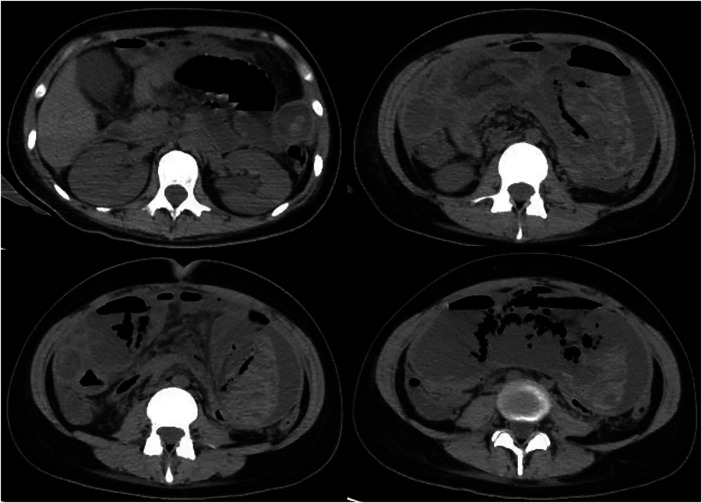
Abdominal CT scans at admission indicating intestinal obstruction.

**Figure 2:**
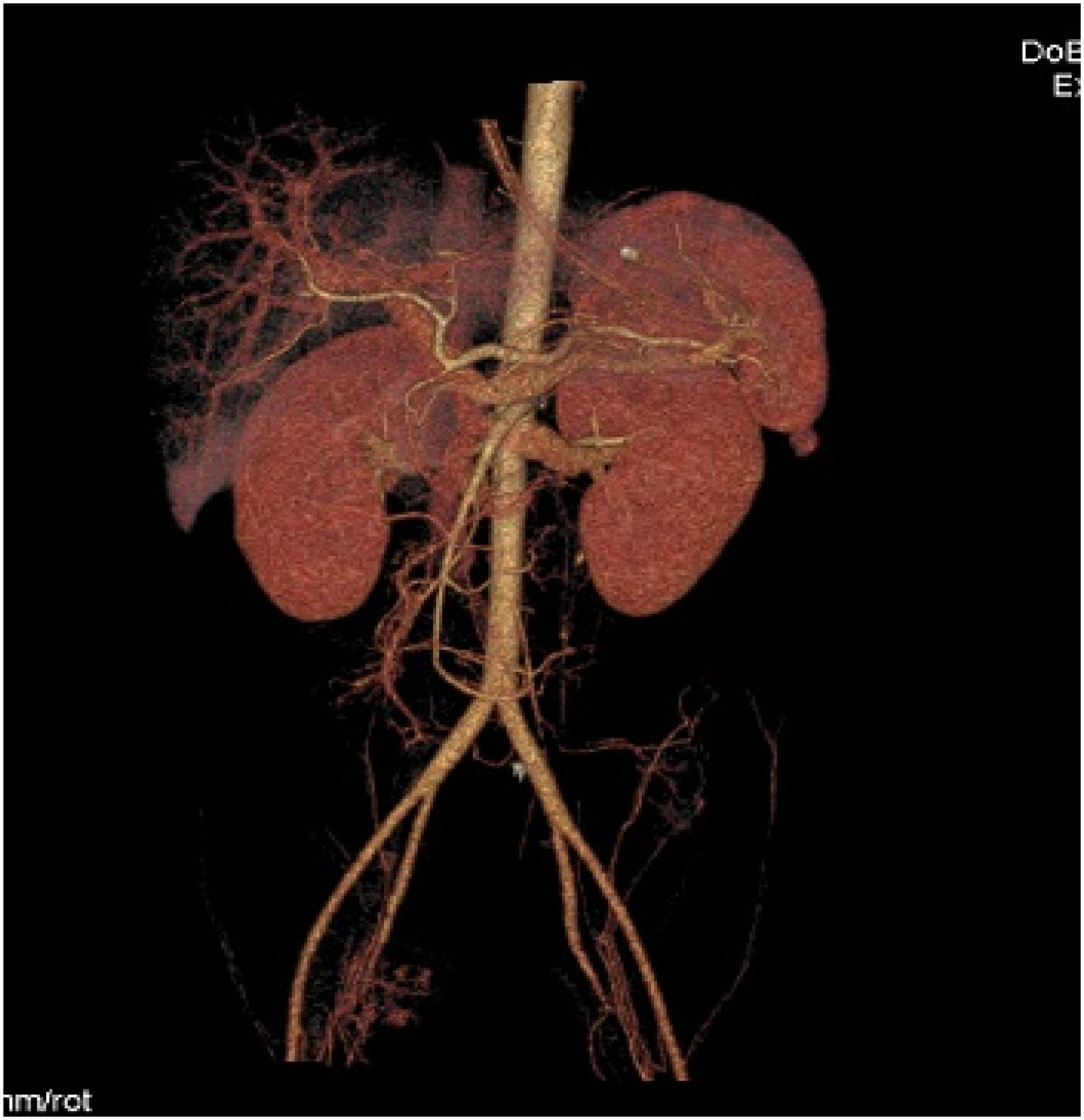
Ct mesenteric arteriography and venography.

On August 6, 2024, at our hospital, she underwent chest and abdominal CT scans, which revealed incomplete small bowel obstruction and mild ascites (Figure 1). On the same day, CT mesenteric arteriography and venography did not show any obvious abnormality in the abdominal aorta, the superior and inferior mesenteric arteries, or the superior mesenteric veins (Figure 2).

At our admission, the patient received supportive treatment, including acid suppression, hepatoprotection, diuretics, antibiotics, anticoagulation, improvement of intestinal perfusion, microcirculation enhancement, blood transfusion, fluid resuscitation, and correction of electrolyte imbalances. On August 8, 2024, interventional mesenteric angiography was performed and showed the absence of thrombosis in the mesenteric arteries and veins. Thus, anticoagulant therapy was discontinued, and then, the patient further received octreotide. Despite aggressive pharmacological therapy was administered, the patient still had persisted abdominal pain without significant improvement. Thus, exploratory laparotomy was performed after obtaining the written informed consents from her family.

**Figure 3:**
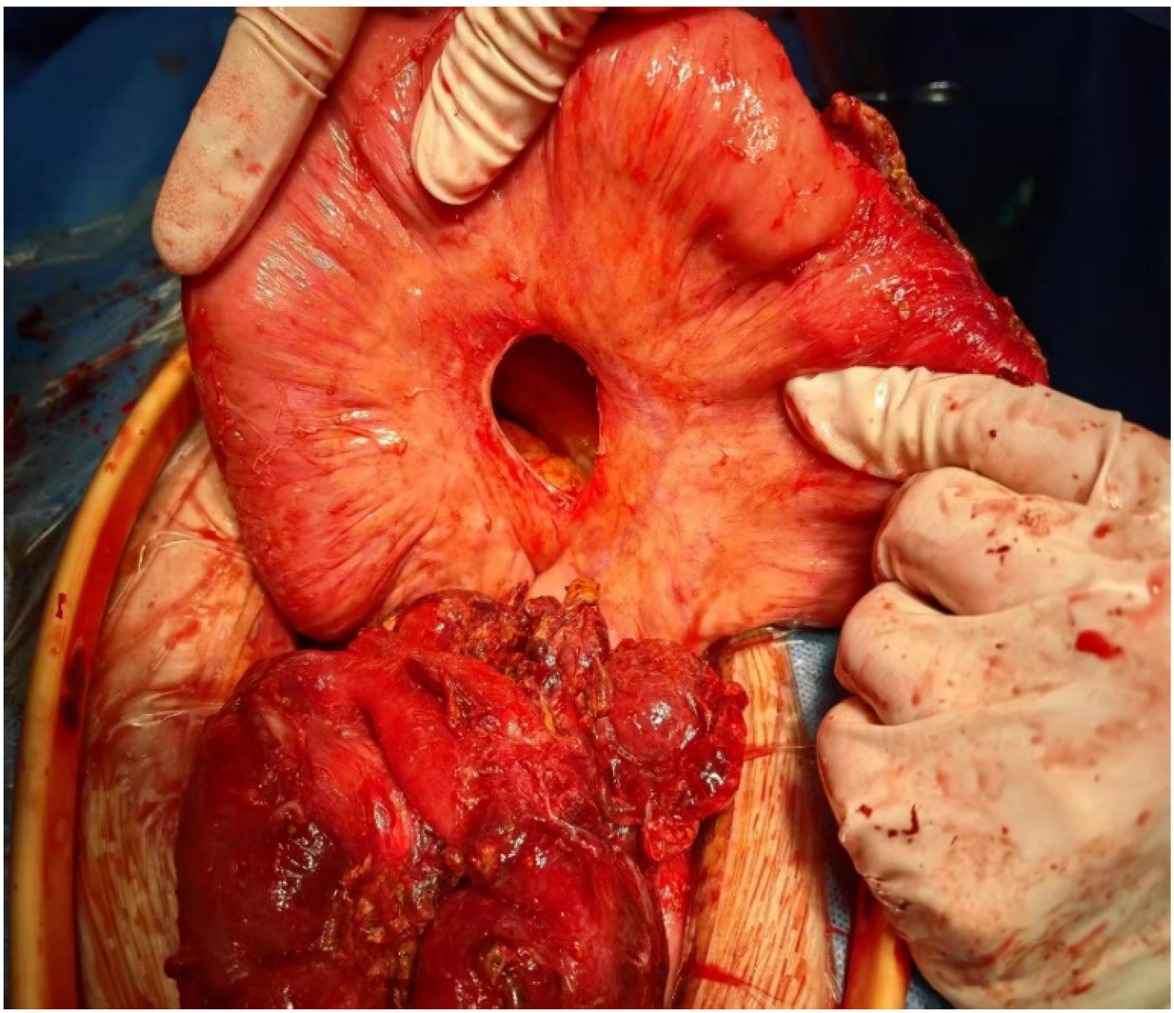
Laparotomy demonstrating a mesenteric defect with a diameter of approximately 3 cm in the small bowel mesentery about 40 cm distal to the treitz ligament. A segment of the small intestine had herniated through this defect, forming an internal abdominal hernia.

Intraoperatively, a mesenteric defect with a diameter of approximately 3 cm was identified at a site 40 cm distal to the Treitz ligament (Figure 3). Small intestine had herniated through this mesenteric defect, forming an internal abdominal hernia. Considering her prolonged course, the herniated bowel had become obstructed, ischemic, and strangulated. Starting at approximately 130 cm distal to the Treitz ligament, the small bowel was necrotic, forming an “intestinal mass” making it impossible to determine the exact length of necrosis. The distal ileum, approximately 15 cm proximal to the ileocecal junction, remained viable. The necrotic segment was resected, and the distal ileal stump was closed. A proximal ileostomy was created through a separate incision in the right lower quadrant. After surgery, the patient was transferred to the intensive care unit.

**Figure 4:**
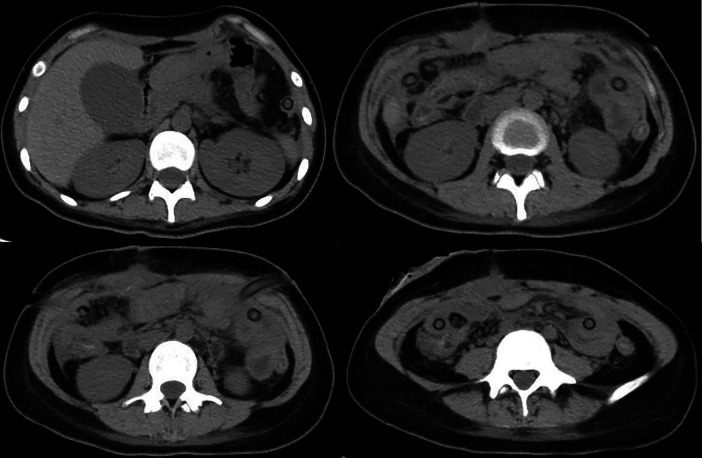
Postoperative abdominal CT scans showing no intestinal dilation.

Postoperatively, the patient was given comprehensive supportive care, including mechanical ventilation, anti-inflammatory therapy, and organ function protection. Her vital signs remained stable, and she was successfully weaned from mechanical ventilation on postoperative day 5. She was transferred to the general ward on postoperative day 14 (Figure 4). On postoperative day 17, the patient resumed oral intake, after the removal of intra-abdominal drainage catheter. She was discharged one month after surgery. During a three-month follow-up period, she was well without abdominal complaints.

## 2 Discussion

Internal hernias are relatively rare in patients without a history of abdominal surgery, with a prevalence of only 0.2%–0.9% in autopsy studies (1–3). Around 4.1% of intestinal obstruction and necrosis are attributable to internal hernias; however, they are frequently overlooked in the differential diagnosis during clinical evaluation (4–6). Currently, the diagnostic value of abdominal CT scans in acute abdomen is gradually highlighted. If CT scans show abnormal clustering of small bowel loops proximal to the obstruction, distortion or crowding of mesenteric vessels, and characteristic imaging signs, such as the “whirl sign,” “comb sign,” “stranding sign,” and “target sign” (3, 7–11), internal hernia should be suspected.

Our patient mainly presented with acute abdominal pain, but did not undergo surgical intervention in a timely fashion due to her family's concerns about surgical risks. The patient experienced transient symptomatic improvement following aggressive conservative medical management, resulting in clinical misjudgment. Traditionally, the presence of peritoneal irritation signs, fever, and high white blood cell levels are indicative of strangulated obstruction in patients with intestinal obstruction and necrosis (12). However, as proposed by some researchers, there was no statistically significant difference in peritoneal signs, body temperature, white blood cell count, or neutrophil percentage between patients with and without intestinal necrosis (13–15). Moreover, some patients with severe internal hernias may not have any sign of peritoneal irritation (16–18). This may be related to the rapid onset and progression of acute intestinal obstruction caused by internal abdominal hernia, which can lead to intestinal ischemia within a short period. Since the metabolic activity of the intestinal mucosa is higher than that of the serosa layer, necrosis initially occurs in the mucosal layer before the involvement of parietal peritoneum in patients with intestinal ischemia (19). As a result, there are no typical peritoneal sign at early stages of the disease.

Initially, this patient was considered to have intestinal obstruction and necrosis caused by ischemic bowel diseases, but they were excluded after clinical and imaging examinations. Since her symptoms were not relieved by conservative medical treatment, she underwent laparotomy after we sufficiently communicated with her family. Expect for the hesitation from the patient's family members, this delayed decision to laparotomy was also caused by several other factors, including: 1) the patient had no prior history of abdominal surgery, and internal abdominal hernia is an inherently rare condition; 2) due to the lack of typical CT findings, the diagnosis could not be established before surgery; and 3) the patient experienced temporary improvement, which led to the misjudgment that conservative treatment would remain effective. Internal hernias are characterized by insidious and nonspecific clinical symptoms, posing a challenge to early diagnosis (20). At late stage of the disease, intermittent obstruction develops, and even acute intestinal obstruction. In the presence of bowel incarceration, patients may rapidly develop exudation, necrosis, and even peritonitis. Although CT images can provide a relatively accurate assessment of anatomical structures and vascular supply associated with internal hernia at the late stages of the disease, the optimal timing of surgical intervention is often missed. Despite surgery was life-saving and successful in our case, extensive small bowel necrosis was observed intraoperatively, causing postoperative short bowel syndrome. This case highlights that immediate laparotomy, rather than wait-and-see, should be considered even in the absence of typical imaging features, if intestinal obstruction fails to respond to conservative treatment.

## 3 Conclusion

As a rare and atypical cause of acute abdomen, internal abdominal hernia poses a diagnostic challenge, necessitating the heightened alertness to monitor disease progression. Simultaneously, early multidisciplinary collaboration and timely intervention are crucial to improve the prognosis.

## Data Availability

The original contributions presented in the study are included in the article/Supplementary Material, further inquiries can be directed to the corresponding authors.
